# Cortical microtubule rearrangements and cell wall patterning

**DOI:** 10.3389/fpls.2015.00236

**Published:** 2015-04-08

**Authors:** Yoshihisa Oda

**Affiliations:** ^1^Center for Frontier Research, National Institute of Genetics, Mishima, Japan; ^2^Department of Genetics, The Graduate University for Advanced Studies, Mishima, Japan; ^3^Precursory Research for Embryonic Science and Technology, Japan Science and Technology Agency, Kawaguchi, Japan

**Keywords:** cytoskeleton, ROP GTPase, MAPs, gamma-tubulin complex, katanin, MIDD1

## Abstract

Plant cortical microtubules, which form a highly ordered array beneath the plasma membrane, play essential roles in determining cell shape and function by directing the arrangement of cellulosic and non-cellulosic compounds on the cell surface. Interphase transverse arrays of cortical microtubules self-organize through their dynamic instability and inter-microtubule interactions, and by branch-form microtubule nucleation and severing. Recent studies revealed that distinct spatial signals including ROP GTPase, cellular geometry, and mechanical stress regulate the behavior of cortical microtubules at the subcellular and supercellular levels, giving rise to dramatic rearrangements in the cortical microtubule array in response to internal and external cues. Increasing evidence indicates that negative regulators of microtubules also contribute to the rearrangement of the cortical microtubule array. In this review, I summarize recent insights into how the rearrangement of the cortical microtubule array leads to proper, flexible cell wall patterning.

## Introduction

Plant cells are enclosed by cell walls composed of cellulose, hemicellulose, pectin, and lignin. Plant organ and tissue growth involves dramatic cellular expansion, the direction of which is strictly controlled by the deposition patterns of the physically rigid cellulose microfibrils. Cell wall patterning is regulated in a flexible manner in response to various external signals such as gravity, light, water, and nutrients, which alter the direction of shoot and root growth. In addition to growth patterns, the deposition patterns of cell walls are elaborately regulated in differentiating cells, such as pavement cells and xylem cells. In differentiating xylem vessels, lignified water-impermeable secondary cell walls are locally deposited, forming distinct patterns on the plasma membrane (such as annular, spiral, reticulate, and pitted patterns) to allow xylem sap transport between the vessels and to prevent vessel collapse under the negative pressure of transpiration. Proper patterning of cell walls is, therefore, essential for determining both cell shape and function.

Plant cortical microtubules form a non-centrosomal array that is laterally anchored to the plasma membrane, regulating the targeting of the cellulose synthase complex to the plasma membrane (Gutierrez et al., 2009; Crowell et al., 2011; Sampathkumar et al., 2013) and the trajectory of this complex (Paredez et al., 2006) through the action of cellulose synthase-interacting proteins (Bringmann et al., 2012; Li et al., 2012). Rearrangement of cortical microtubules immediately influences the trajectory of the cellulose synthase complex (Paredez et al., 2006; Chan et al., 2010). Recent studies suggest that cortical microtubules recruit exocytotic vesicles (Oda et al., 2014) and direct the deposition of non-cellulosic materials (Zhu et al., 2015). Cortical microtubule arrays thus play essential roles in regulating the deposition patterns of both cellulosic and non-cellulosic cell wall materials.

In vegetative organs such as roots, hypocotyls, stems, and petioles, most bipolar cells develop transversely co-aligned cortical microtubules to enable their anisotropic growth. However, plant cells also exhibit dynamic rearrangements of cortical microtubules such as rotation (Lloyd, 2011), reorientation (Shaw, 2013), global disassembly (Wang et al., 2007), and reorganization into distinct patterns (Oda and Fukuda, 2012b, 2013a,c). Recent genetic and live cell imaging studies revealed the dynamic process and molecular mechanism underlying these cortical microtubule rearrangements, as summarized in this review. Before addressing the cortical microtubule rearrangements, I briefly overview the transverse cortical microtubule organization to spot the typical behavior of the cortical microtubules.

## Dynamics and Self-Organization of the Transverse Cortical Microtubule Array

Cortical microtubules elongate at their plus ends via repeated catastrophe and rescue events, while they exhibit slow, continuous depolymerization at their minus ends (Shaw et al., 2003), and nucleate primarily at the plasma membrane, mainly on the side of pre-existing cortical microtubules at an average angle of 40°(resulting in the formation of a branch-form microtubule) or along the mother microtubule (resulting in the formation of parallel microtubules; Murata et al., 2005; Chan et al., 2009). Growing microtubules often interact with other cortical microtubules; the meeting of microtubules at a steep angle induces catastrophe, while that at a shallow angle induces bundling (Dixit and Cyr, 2004). In addition, cortical microtubules are preferentially severed near the nucleation site and crossing point.

Recent genetic and biochemical studies in *Arabidopsis* revealed that normal nucleation of cortical microtubules requires an intact gamma-tubulin ring complex, comprising gamma-tubulin, six gamma-tubulin complex proteins (GCPs), and their putative regulatory proteins including Augmin complex and a B″ subunit of protein phosphatase 2A (PP2A), TON2 Defects in these components affect the frequency and geometry of cortical microtubule nucleation, resulting in a hyper-parallel microtubule array (Nakamura and Hashimoto, 2009; Kong et al., 2010; Kirik et al., 2012; Nakamura et al., 2012; Liu et al., 2014; Walia et al., 2014).

Microtubule severing appears to solely depend on KTN1, a katanin p60 subunit (Wightman and Turner, 2007; Nakamura et al., 2010; Lindeboom et al., 2013b; Wightman et al., 2013; Zhang et al., 2013). Loss of *KTN1* dramatically reduces the frequency of microtubule severing, weakens co-alignment of cortical microtubules, and delays or abolishes various rearrangements of cortical microtubules (see below). Two proteins regulate the activity of KTN1: RIC1 and SPR2. RIC1 is an effector of ROP6 GTPase, which activates KTN1 to promote parallel ordering of cortical microtubules (Lin et al., 2013). By contrast, SPR2, a microtubule-associated protein (MAP), accumulates at the microtubule crossing point to prevent severing by KTN1, allowing non-ordered cortical microtubules to persist (Wightman et al., 2013). These findings suggest that KTN1 activity is precisely controlled in the cell.

Genetic studies and computer simulations predicted that these dynamic properties of cortical microtubules are sufficient to enable self-organization of globally co-aligned microtubule within a cell (Dixit and Cyr, 2004; Wasteneys and Ambrose, 2009; Eren et al., 2010; Tindemans and Mulder, 2010; Tindemans et al., 2010; Ambrose et al., 2011; Deinum et al., 2011). However, recent studies revealed that various signals regulate microtubule behavior to define the orientation, density, and heterogeneity of cortical microtubule organization at the subcellular and supercellular level.

## Reorientation: Transverse to Longitudinal

In hypocotyl and root epidermal cells, dynamic reorientation of the cortical microtubule array can occur in response to light or hormone application to inhibit cell expansion. Auxin treatment induces reorientation of cortical microtubules from transverse to longitudinal in *Arabidopsis* root and hypocotyl epidermis. This auxin-induced reorientation of cortical microtubules requires, ROP6 GTPase, its effector protein RIC1, and KTN1 (Chen et al., 2014). Since auxin application affects the direction of cortical microtubules within minutes, this pathway is likely a non-transcriptional response (Chen et al., 2014). In leaf epidermis, auxin activates ROP6 via TMK transmembrane kinase (Xu et al., 2014). ROP6, in turn, promotes microtubule severing by KTN1 through the action of RIC1 (Lin et al., 2013). Similarly, auxin may activate KTN1 through ROP6 and RIC1 to promote the reorientation of cortical microtubules. The auxin binding protein ABP1 was suggested to mediate this auxin signaling to the ROP6-RIC1-KTN1 pathway (Chen et al., 2014; Xu et al., 2014). However, it was recently demonstrated that ABP1 is not required for normal auxin response (Gao et al., 2015). Further investigation is needed to reveal the molecular pathway from auxin to the ROP signaling.

**FIGURE 1:**
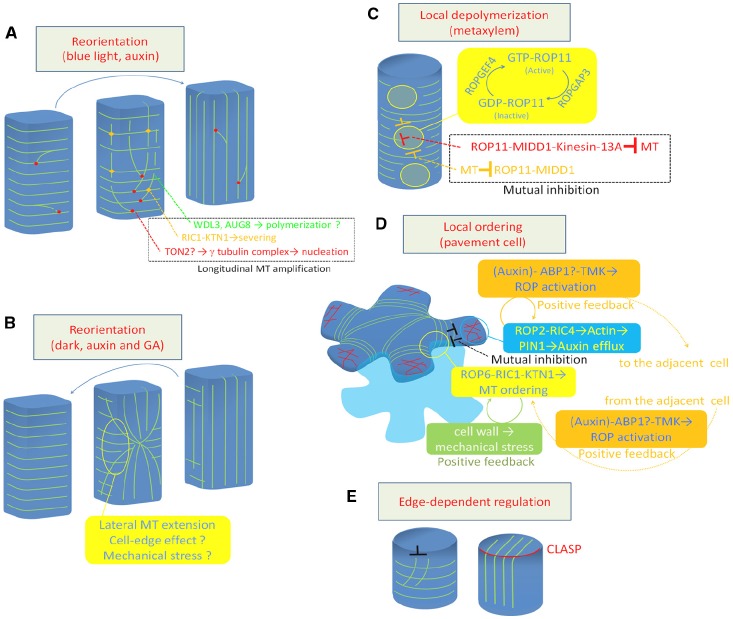
**Regulation of cortical microtubule rearrangements. (A)** Reorientation from transverse to longitudinal. **(B)** Reorientation from longitudinal to transverse. **(C)** Local depolymerization in xylem vessel cells. **(D)** Local ordering in leaf pavement cell. **(E)** Cell edge-dependent regulation by CLASP protein. Green lines indicate cortical microtubules **(A–E)**. Red lines in **(D)** indicate actin microfilaments. MT, microtubule; GA, gibberellic acid.

The behavior of microtubules was precisely analyzed during blue light-triggered reorientation (from transverse to longitudinal) in the hypocotyl epidermis (Lindeboom et al., 2013b). Blue light irradiation temporally increases the frequency of severing of longitudinally growing microtubules at the microtubule crossing point. The basal fragment of the severed microtubules is then rescued at high frequency to restart its growth, resulting in a significant amplification of longitudinal microtubules (Figure 1A). Blue light signaling may activate severing activity or targeting of KTN1 as this efficient reorientation of cortical microtubules is delayed in both *ktn1* and *phot1/phot2* double mutants (Lindeboom et al., 2013b).

Whether microtubule nucleation is involved in the regulation of microtubule reorientation is an interesting issue. Blue light-triggered microtubule reorientation is not efficiently induced in the *ton2* mutant, in which the nucleation mode is shifted from branch to parallel (Kirik et al., 2012). Furthermore, *phot1/phot2* mutants exhibit higher frequencies of parallel nucleation than wild type (Lindeboom et al., 2013b). These findings indicate that blue light signaling positively regulates branch nucleation as well as microtubule severing.

Positive regulation of microtubule polymerization and stability is also important for blue light-triggered microtubule orientation. Loss of *AtAUG8* prevents cortical microtubule reorientation from transverse to longitudinal (Cao et al., 2013). AtAUG8 localizes to the plus end of cortical microtubules and promotes polymerization of microtubules *in vitro* (Cao et al., 2013), suggesting that AtAUG8-derived microtubule polymerization is required for microtubule reorientation.

Another MAP, WDL3, a member of WAVE-DAMPER2 (WVD2) family, accelerates light-triggered reorientation of cortical microtubules, while knockdown slows reorientation (Liu et al., 2013). WDL3 promotes bundling and stabilization of microtubules *in vitro*. Importantly, WDL3 is preferentially degraded in the dark, while light irradiation promotes the accumulation of this protein, suggesting that light signaling positively regulates microtubule stabilization by preventing the degradation of WDL3. These positive regulators may contribute to the active amplification of longitudinal microtubules.

## Reorientation: Longitudinal to Transverse

Cortical microtubule reorientation from longitudinal to transverse can be induced in the hypocotyl epidermis by hormone or dark treatment. Several MAPs were found to influence the reorientation (Li et al., 2011; Qin et al., 2012; Wang et al., 2012). However, the behavior of cortical microtubules during this reorientation was only recently elucidated.

Vineyard et al. (2013) conducted time-lapse observations of longitudinal-to-transverse reorientation of cortical microtubules triggered by auxin and gibberellin treatment. They found that the longitudinal array was replaced by a transverse array from the outer anticlinal side walls at the mid zone. The transverse array area then gradually expanded toward both sides of the apical and basal ends, suggesting that the reorientation of cortical microtubules is likely driven by a progressive change in direction of microtubule polymerization initiating in the anticlinal side walls (Vineyard et al., 2013; Figure 1B).

Atkinson et al. (2014) carefully examined the nucleation mode during reorientation triggered by auxin and gibberellin treatment. They found that the microtubule nucleation frequency and branch-to-parallel nucleation ratio are not altered during this process, even in the *ton2* mutant, which slowly undergoes cortical microtubule reorientation, suggesting that reorientation does not depend on changes in the microtubule nucleation mode (Atkinson et al., 2014).

Sambade et al. (2012) also observed a transition of cortical microtubules from longitudinal to transverse in hypocotyl epidermis in response to dark treatment. They found that cortical microtubules are reoriented through the formation of star-like structures, where cortical microtubules run radially from the center of the periclinal side wall. Computer simulations suggest that the formation of such structures can be attributed to the reduced frequency in which microtubules can elongate through the steep edge between the anticlinal side face and the periclinal outer face (Sambade et al., 2012).

These studies address the complexity of cortical microtubule organization in different faces. Indeed, the behavior of the cortical microtubule array differs between the outer and inner surface. Cellulose microfibrils also exhibit independent microtubule deposition patterns in each face (Chan et al., 2010, 2011; Crowell et al., 2011). These facts suggest that regulation of microtubule behavior at the cell edge may contribute to the global organization of cortical microtubules (see below). Furthermore, Sambade et al. (2012) found that the dark-treated hypocotyl starts cell growth slightly prior to cortical microtubule reorganization. This suggests another possibility that mechanical stress along each cell face may one of the driving forces to reorient cortical microtubules.

## Global Destabilization of Cortical Microtubules

While osmotic and salt stresses induce the disappearance of cortical microtubules, negative regulators of microtubule formation in plants were only recently identified. PHS1, an atypical kinase, was recently shown to regulate cortical microtubule destabilization in response to osmotic and salt stresses (Naoi and Hashimoto, 2004; Walia et al., 2009; Pytela et al., 2010; Fujita et al., 2013). PHS1 is a hybrid protein harboring a tubulin kinase domain and an inhibitory phosphatase domain. Under normal conditions, the tubulin kinase domain of PHS1 is inactivated by MAPK kinases (Pytela et al., 2010). However, once the cell perceives salt or osmotic stress, the phosphatase domain of PHS1 unmasks the tubulin kinase, allowing phosphorylation of alpha tubulin. This process leads to the production of polymerization-incompatible tubulin, which in turn leads to global disassembly of cortical microtubules (Fujita et al., 2013).

## Local Destabilization of Cortical Microtubules

Recent studies using *Arabidopsis* xylogenic cell culture revealed that depolymerization of cortical microtubules is regulated at the subcellular level to form the pitted pattern of secondary cell walls (Figure 1C). In differentiating xylem cells, ROP11 GTPase is locally activated at the plasma membrane to recruit the microtubule-binding protein MIDD1 (Oda and Fukuda, 2012a). MIDD1 then recruits Kinesin-13A, an ATP-dependent microtubule depolymerizing kinesin, to induce depolymerization of cortical microtubules (Oda et al., 2010; Oda and Fukuda, 2013b). The local activation of ROP11 appears to be cell-autonomously controlled by ROPGEF4 and ROPGAP3 (a ROP guanine nucleotide exchange factor and a ROP GTPase activating protein, respectively). In addition, cortical microtubules surrounding the ROP-activated domain enclose activated ROP11 through MIDD1 (Oda and Fukuda, 2012a). This mutual inhibition between the plasma membrane domain and the surrounding cortical microtubules appears to strictly control local microtubule disassembly.

## Local Ordering of Cortical Microtubules

Another example of local regulation of cortical microtubule behavior was studied in the leaf epidermis, where pavement cells develop lobes and indentations reciprocally to form an interdigitation. In developing pavement cells, microtubules are locally ordered to restrict cell expansion, resulting in indentation (Figure 1D). This local ordering of cortical microtubules requires local accumulation of ROP6 GTPase (Fu et al., 2005), which recruits RIC1 to activates KTN1 (Lin et al., 2013).

Unlike in xylem cells, the contribution of ROPGEFs and ROPGAPs to this local activation of the ROP6-RIC1-KTN1 pathway is currently unclear. Instead, other two distinct mechanisms appear to maintain this local microtubule regulation. In developing leaf pavement cells, ROP2 also accumulates in space between ROP6-containing indentations. ROP2 recruits the effector protein RIC4 to induce actin microfilament assembly, which promotes cell outgrowth. This ROP2-RIC4-actin pathway mutually inhibits the ROP6-RIC1-microtubule pathway (Fu et al., 2005). At the same time, the accumulated actin microfilaments inhibit endocytosis, leading to accumulation of the auxin efflux carrier PIN1 at the plasma membrane (Nagawa et al., 2012). PIN1, in turn, promotes auxin accumulation in the apoplastic space, which further activates ROP2 and ROP6 in the adjacent cell through TMK kinases, forming an positive feedback loop (Xu et al., 2010, 2014). These regulatory mechanisms explain how plant cells coordinate subcellular and intercellular spatial signaling.

## Cell Edge-Based Regulation of Cortical Microtubule Arrays

Recent studies revealed an emerging function of cell edges, i.e., a distinct, steep geometry within the cell. During the early G1 phase, GCP2, and GCP3 are temporally localized at the edge along the newly formed end wall. Cortical microtubules are nucleated from the cell edge to form a longitudinal array, which is then replaced with a transverse array (Ambrose and Wasteneys, 2011; Lindeboom et al., 2013a), demonstrating that the cell edge plays a distinct role in cortical microtubule organization.

Another function of the cell edge involves CLASP, a plasma membrane-bound MAP that plays crucial roles in cell polarity, cell division, and cell shaping (Ambrose et al., 2007, 2011, 2013; Ambrose and Wasteneys, 2008; Pietra et al., 2013). The loss-of-function *clasp* mutant contains hyper-parallel cortical microtubule arrays, which is probably due to the high frequency of catastrophe that occurs when microtubules encounter the steep cell edge. By contrast, in wild-type plants, intact CLASP at the cell edge allows cortical microtubule to elongate through the steep cell edge, forming transfacial bundles (Ambrose et al., 2011; Figure 1E). This local regulation of cortical microtubule behavior is assumed to ensure the global and flexible organization of cortical microtubules in the polygonal cell geometry.

## Mechanical Stress Regulates Cortical Microtubule Ordering at both the Subcellular and Supercellular Levels

In addition to MAPs and ROP signaling, mechanical stress also regulates cortical microtubule organization. When plant tissues or organs are placed under artificial pressure, the cortical microtubules become co-aligned along the direction of the stress, suggesting that plant cells can regulate cortical microtubule organization according to the stress (Hamant et al., 2008; Heisler et al., 2010; Uyttewaal et al., 2012; Sampathkumar et al., 2014). In anisotropically growing organs, tissue-embedded cells are assumed to be under anisotropic tension derived from the tight attachment with the surrounding cells. Therefore, mechanical stress should influence cortical microtubule organization, even under natural conditions. Indeed, cell ablation and cell wall digestion (which is thought to rearrange the tensile pressure pattern) induce rearrangement of the cortical microtubule array along the direction of newly formed tensile pressure (Hamant et al., 2008; Heisler et al., 2010; Sampathkumar et al., 2014).

At the subcellular level, mechanical stress activates KTN1 to promote cortical microtubule ordering, which reinforces the cell wall by directing cellulose deposition. The cell wall, in turn, produces further mechanical stress, resulting in positive feedback of the microtubule arrangement (Sampathkumar et al., 2014). Importantly, mechanical stress also regulates PIN1 polarization through a partially common pathway with cortical microtubules (Heisler et al., 2010). Therefore, mechanical stress may influence microtubule organization and auxin flow to coordinate cell growth in tissue and organ development.

## Conclusion and Perspectives

Increasing evidence suggests that various spatial signaling regulate the rearrangement of cortical microtubules by modulating the self-organized transverse array. The typical behavior of cortical microtubules, including severing, nucleation, depolymerization, and polymerization are regulated at subcellular and supercellular level to promote the rearrangement of cortical microtubule array. ROP GTPase signaling, CLASP, and mechanical signals obviously bias these microtubule behavior in the cell. Rearrangement of cortical microtubules by these spatial signaling alters the cell wall deposition pattern, which in turn influences the behavior of cortical microtubules through the mechanical signals. It is still elusive how mechanical signals influence the behavior of cortical microtubule. Considering that mechanical signal requires katanin for microtubule rearrangement and that ROP GTPase activates katanin through RIC1, mechanical signals may regulate cortical microtubule array through ROP signaling at the plasma membrane. Mechanical signaling also regulates auxin flow in microtubule-independent manner, which can activate ROP GTPases. Therefore, cortical microtubule rearrangements and cell wall patterning form a complex circuit through mechanical signals, ROP, and auxin. ROP signaling, that can be cell-autonomously established by ROPGEF and ROPGAP, must be one of the initiators of the regulatory circuit. Elucidating how the ROP signals are *de novo* organized is critical for further understanding of the cortical microtubule rearrangement and cell wall patterning.

### Conflict of Interest Statement

The author declares that the research was conducted in the absence of any commercial or financial relationships that could be construed as a potential conflict of interest.
